# Supplementary material for: Bright single photon emitters with enhanced quantum efficiency in a two-dimensional semiconductor coupled with dielectric nano-antennas
Source: Nat Commun. 2021 Oct 18;12:6063. doi: 10.1038/s41467-021-26262-3 (PMC8523570; doi:10.1038/s41467-021-26262-3)
Supplement: Supplementary file 1 — Supplementary Information [file 41467_2021_26262_MOESM1_ESM.pdf]

# Supplementary information for: Bright single-photon emitters with enhanced quantum efficiency in a two-dimensional semiconductor coupled with dielectric nano-antennas

Luca Sortino,[1,2] Panaiot G. Zotev,[1] Catherine L. Phillips,[1] Alistair J. Brash,[1] Javier Cambiasso,[3] Elena Marensi,[4] A. Mark Fox,[1] Stefan A. Maier,[2,3] Riccardo Sapienza,[3] and Alexander I. Tartakovskii[1]

[1]*Department of Physics and Astronomy, University of Sheffield, Sheffield, S3 7RH, United Kingdom*

[2]*Chair in Hybrid Nanosystems, Nanoinstitute Münich, Faculty of Physics, Ludwig-Maximilians-Universität München, 80539 Münich, Germany*

[3]*The Blackett Laboratory, Department of Physics, Imperial College London, London, SW7 2BW, United Kingdom*

[4]*IST Austria, Am Campus 1, 3400 Klosterneuburg, Austria*

# SUPPLEMENTARY NOTE I: NUMERICAL SIMULATIONS OF THE PHOTOLUMINESCENCE ENHANCEMENT FACTOR

The PL intensity collected from a single dipole emitter coupled to an optically driven nano-antenna is highly dependent on its relative position and orientation in respect to the scattered field, defined by the vector $\mathbf{r}$, and originates from three factors [1]:

$$I(\mathbf{r}, \lambda_{\text{em}}) \propto \gamma_{\text{exc}}(\mathbf{r}, \lambda_{\text{exc}}) \cdot QE(\mathbf{r}, \lambda_{\text{em}}) \cdot \eta_{\text{NA}}(\mathbf{r}, \lambda_{\text{em}}) \tag{1}$$

The relative PL enhancement factor $\langle EF \rangle$ [2] is given by the ratio between PL intensity values obtained when the dipole is placed on GaP nano-antennas and on $SiO_2$ nano-pillars, $\langle EF \rangle = I_{\text{GaP}}/I_{\text{SiO}_2}$. We carried out a set of numerical finite-difference time-domain (FDTD) simulations for an in-plane dipole emitting at $\lambda_{\text{em}} = 750$ nm, placed either on top of GaP dimer nano-antennas, or on $SiO_2$ nano-pillars on a $SiO_2$(100 nm)/Si substrate. The heights of the GaP dimers and the $SiO_2$ pillars were set to 200 nm and 100 nm, respectively, matching the structures used in our experiments. In our simulations we find that by increasing the height of $SiO_2$ pillars, a negligible effect on their optical properties is observed due to the lack of Mie optical resonances.

The first factor in Eq.1, $\gamma_{\text{exc}}$, describes the increased absorption cross section, dependent on the local electric field intensity $(|E|/|E_0|)^2$, where $|E|^2$ is the intensity of the scattered radiation and $|E_0|^2$ the intensity of the normally incident linearly polarized plane wave. Supplementary Fig.1 shows the maxima of the local E field at the top surface of the nano-antenna ($z = 200$ nm) taken along the line connecting each nano-pillar centre (see Inset). In Supplementary Fig.1b we show the maximum value of the field on GaP dimers (red) and on $SiO_2$ nano-pillars (blue). Their ratio (yellow) defines the relative field enhancement, equal to $\gamma_{\text{exc}}$.

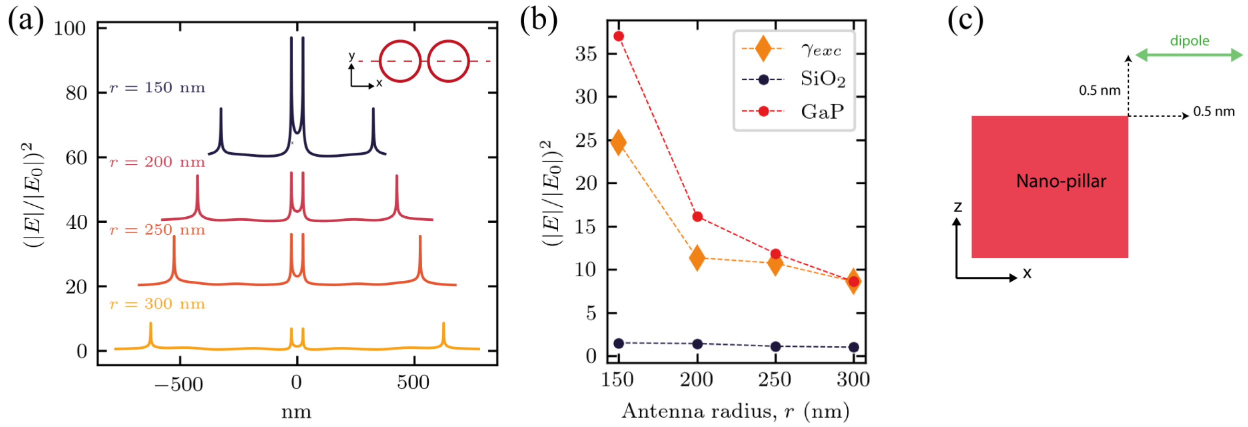


SUPPLEMENTARY FIGURE 1. (a) Profiles of the electric field intensity, $(|E|/|E_0|)^2$, at the top surface of the nano-antenna (z=200 nm) under a plane wave excitation at $\lambda_{\text{exc}} = 638$ nm linearly polarized along the x-axis. The profile is taken along the axis connecting each nano-pillar centre (dashed line in Inset). The profiles are shifted vertically for display purposes. (b) Values of the maxima of the electric field intensity for GaP dimer nano-antennas (red), $SiO_2$ nano-pillars (blue) and their ratio $\gamma_{\text{exc}} = \gamma_{\text{exc}}^{\text{GaP}}/\gamma_{\text{exc}}^{\text{SiO}_2}$ (orange). (c) Position of the dipole emitter relative to the nano-pillar edge used in the Purcell factor simulations.

The second factor $QE$ is the quantum efficiency of the dipole, defined as $QE = \gamma_{\rm r}/(\gamma_{\rm r} + \gamma_{\rm nr})$, where $\gamma_{\rm r}$ is the radiative decay rate and $\gamma_{\rm nr}$ is the non-radiative decay rate. In our simulations we used an approximation for low $q$ emitters, where the non-radiative decay $\gamma_{\rm nr} \gg F_{\rm p}\gamma_{\rm r}$, and the change in $q$ can be evaluated only from the radiative decay rate enhancement defined by the Purcell factor [2], $F_{\rm P} = \gamma_{\rm r}/\gamma_{\rm r}^0$, defined as the enhancement of the energy dissipation $P/P_0$ in the numerical simulations [3], where $\gamma_{\rm r}^0$ and $P_0$ ar related to the dipole on planar substrate. The dipole is placed 0.5 nm above the surface and 0.5 nm away from the nano-pillar edge, as shown in Supplementary Fig.2c. The values obtained are shown in the main text in Fig.1c, where they are normalized over the same dipole placed on the flat substrate as reference.

The last factor in Eq.1, $\eta_{\rm NA}$, defines the fraction of the light collected by the numerical aperture ($NA$) of the objective, calculated as the fraction of power emitted in the upwards direction, in a cone defined by the objective $NA$. Supplementary Fig.2a-c shows the radiation pattern for an in-plane dipole ($\lambda_{\rm em} = 750$ nm) placed at the centre of the gap of a GaP dimer nano-antenna (in red) and at the edge of a $SiO_2$ nano-pillar (in grey). In case of GaP nano-antennas, no significant difference was observed when placing the dipole at the edge of the nano-pillar, similarly to $SiO_2$. Due to the higher refractive index, for GaP most of the emitted light is directed downwards into the substrate. For a dipole coupled to GaP dimer nano-antennas we obtained a collection of 7% for antennas with a radius of 300 nm, and up to 10% for $r = 150$ nm. For a dipole on top of a $SiO_2$ nano-pillar, we found a collection from 8% (r = 300 nm) up to 9% (r = 150 nm).

In case of dimer nano-antennas larger than 150 nm we observe a larger enhancement of the collection efficiency and $F_{\rm P}$ compared to the same dipole placed in the gap of the dimer. Following our previously published theoretical calculations [6], this is the point where tensile strain is maximised and thus where the SPEs will likely be positioned. In Supplementary Fig.2d-e we show the effect on the power radiated by a dipole placed at either the gap (purple) or at the outer edges (yellow) of a dimer nano-antenna with a radius of 200 nm.

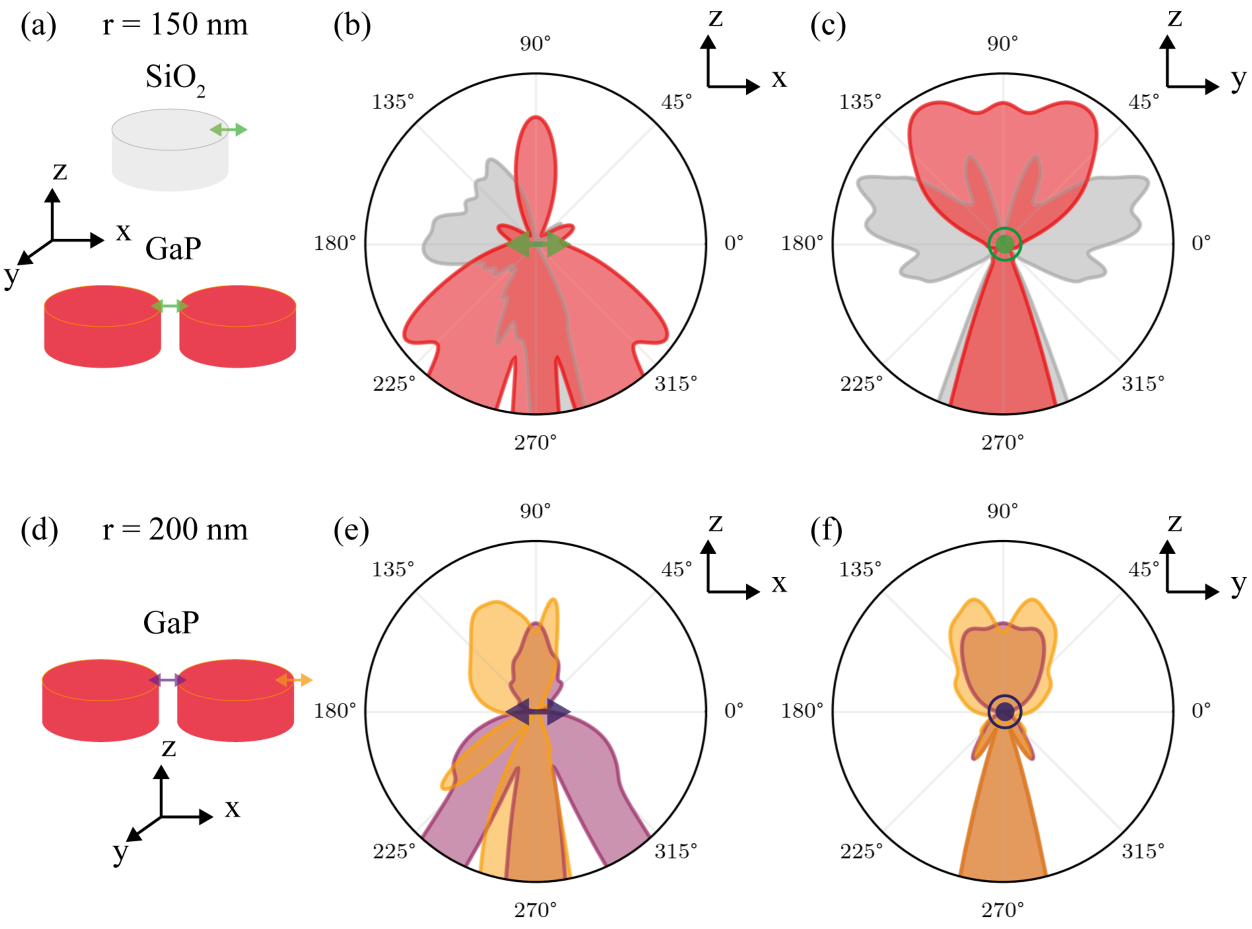


SUPPLEMENTARY FIGURE 2. (a) Schematics of the electric dipole (in green) position and orientation in respect to either a silicon nano-pillar (grey) and a GaP dimer nano-antennas, both with a radius of 150 nm. (b,c) Radiation pattern projection on the $xz$-plane (b) and $yz$-plane (c) for a dipole closely coupled to a $SiO_2$ nano-pillar (in grey) and in the gap of a GaP dimer nano-antenna (in red). (d) Schematics of an electric dipole position and orientation for a GaP dimer nano-antenna with radius of 200 nm. (b,c) Radiation pattern projection on the $xz$-plane (e) and $yz$-plane (f) for a dipole placed in the gap of the dimer (in purple) and at the outer edge (in yellow).

## SUPPLEMENTARY NOTE II: OPTICAL MIE RESONANCES OF GAP DIMER NANO-ANTENNAS

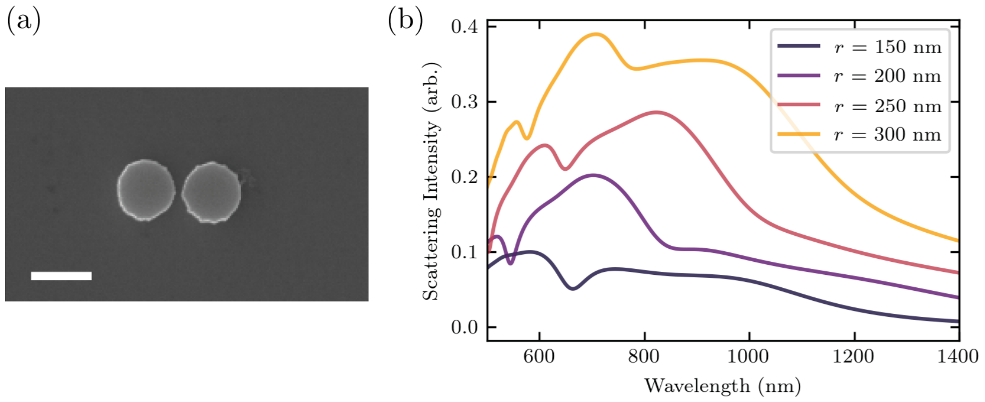


SUPPLEMENTARY FIGURE 3. (a) Electron microscope image of a GaP dimer nano-antenna ($r = 200$ nm, $h = 200$ nm). Scale bar = 200 nm. (b) Simulated scattering cross section for GaP dimer nano-antennas with varying radius $r = 150, 200, 250, 300$ nm, height $h = 200$ nm and gap width $g = 50$ nm)

## SUPPLEMENTARY NOTE III: PHOTOLUMINESCENCE AND POLARIZATION PROPERTIES OF $WSE_2$ SINGLE PHOTON EMITTERS ON GAP NANO-ANTENNAS

Supplementary Fig.4a shows the PL spectrum for a $WSe_2$ monolayer on top of a GaP dimer nano-antenna ($r$ = 250 nm) excited with a linearly polarized laser at 638 nm, and collected under two orthogonal polarization directions (blue and red traces). As shown in Supplementary Fig.4b, the single peak observed in co-polarized collection (highlighted in blue in Fig.S4a) is accompanied by a series of peaks in cross-polarized detection at lower energy (highlighted in red in Fig.S4a). The SPEs on GaP nano-antennas exhibit stable emission over time, as shown in Supplementary Fig.4c, as expected from the improved spectral wandering in highly strained monolayers [4]. As shown in Supplementary Fig.4d, we observe ns PL lifetimes, with faster dynamics for the cross-polarized peaks (red trace $\tau$ = 7 ns) compared to the co-polarized peak (blue trace $\tau$ = 42 ns). The emitters exhibit a similar power saturation behaviour, as shown in Supplementary Fig.4e. In Supplementary Fig.4f we show the homogeneous broadening of the linewidth (FWHM) of the SPE highlighted in

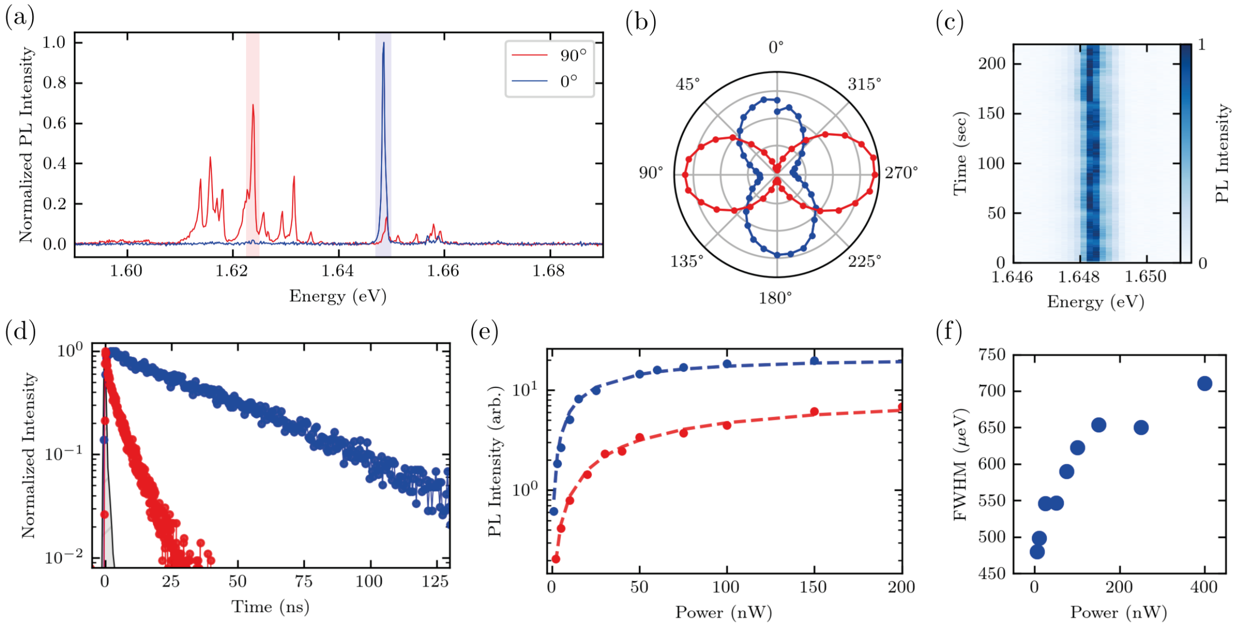


SUPPLEMENTARY FIGURE 4. (a) Monolayer $WSe_2$ PL spectrum, collected on top of a single GaP nano-antenna at a temperature of $T = 4$ K. The PL spectrum in blue is collected with a linear polarizer aligned to the excitation polarization axis, while the PL spectrum in red is recorded after rotating the linear polarizer in the detection path by 90 degrees. (b) Polar plot of the integrated PL intensity, for the peaks highlighted with the same colour in Supplementary Fig.4a. (c) PL emission for the blue peak in Supplementary Fig.4a showing stable emission over time. (d) Time resolved PL dynamics of the peaks highlighted with the same colour in Supplementary Fig.4a. (e) Power dependent saturation of the PL intensity, under a pulsed excitation at 638 nm and 80 MHz repetition rate. A similar PL saturation threshold is found for both types of emitter.(f) Broadening of the linewidth (FWHM) under increasing power density for the co-polarized peak in Supplementary Fig.4a.

blue in Fig.S4a, under increasing excitation power density, obtained by fitting the spectra with a Lorentzian peak function (see Fig.4 in the main text) . Due to the varying anisotropy of the confining potential, SPEs in $WSe_2$ are known to exhibit emission peaks both with and without a fine structure splitting (FSS) at zero magnetic field [5]. As we show in Supplementary Fig.5a-b, we observe the presence of cross polarized peaks with FSS on the order of 600-800 $\mu$eV ( Supplementary Fig.5c) and, on the same nano-antenna, peaks that show a near unity degree of linear polarization with no underlying fine structure. As shown in Supplementary Fig.5d-f, we observed a repeated pattern in the polarization from strain-induced SPEs on top of nano-antennas with different radii. The presence of a bright, high energy peak, usually exhibiting a FSS, is followed by a large number of emitters at lower energies, exhibiting the same polarization axis and no FSS. We ascribe this behaviour to different kinds of strain-induced emitters in the $WSe_2$ layer, related to a different confinement energy and size of the potential well created by nano-scale strain inhomogeneities.

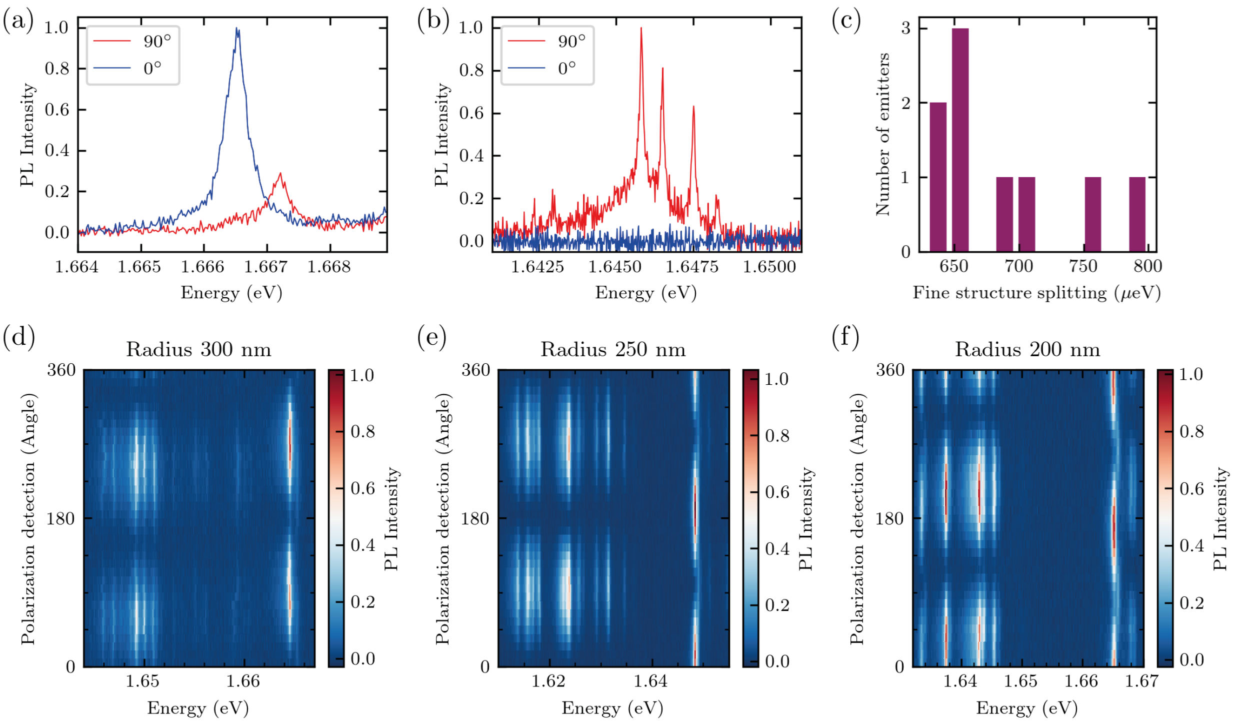


SUPPLEMENTARY FIGURE 5. High resolution PL spectra at co- and cross-polarized detection centered at the blue (a) and red (b) highlighted peaks in Supplementary Fig.4a. Supplementary Fig.5a displays a fine structure split cross-polarized doublet. (b) does not display any FSS and exhibits a near unity degree of linear polarization. (c) Histogram of fine structure splitting values observed for high energy peaks at the positions of different nano-antennas. (d-e) PL emission as a function of the detection polarization angle for localized SPEs on top of GaP dimer nano-antennas with different radii of 300 nm (d), 250 nm (e) and 200 nm (f).

## SUPPLEMENTARY NOTE IV: FABRICATION AND PHOTOLUMINESCENCE PROPERTIES OF $WSE_2$ SINGLE PHOTON EMITTERS ON $SIO_2$ NANO-PILLARS

The $SiO_2$ nano-pillars are fabricated from a thermally grown 290 nm $SiO_2$ layer on a silicon wafer, with radii ranging from 50 to 250 nm, with an electron beam lithography and reactive ion etching system. Supplementary Fig.6a shows an electron microscope image of a resulting $SiO_2$ nano-pillar with a radius of 200 nm and height of 100 nm. The monolayer of $WSe_2$ is transferred onto the $SiO_2$ nano-pillars with an all-dry transfer technique. A bright field image of the transferred monolayer is shown in Supplementary Fig.6b. Supplementary Fig.6c shows the room temperature PL map of the transferred $WSe_2$ monolayer on top of the nano-pillar array.

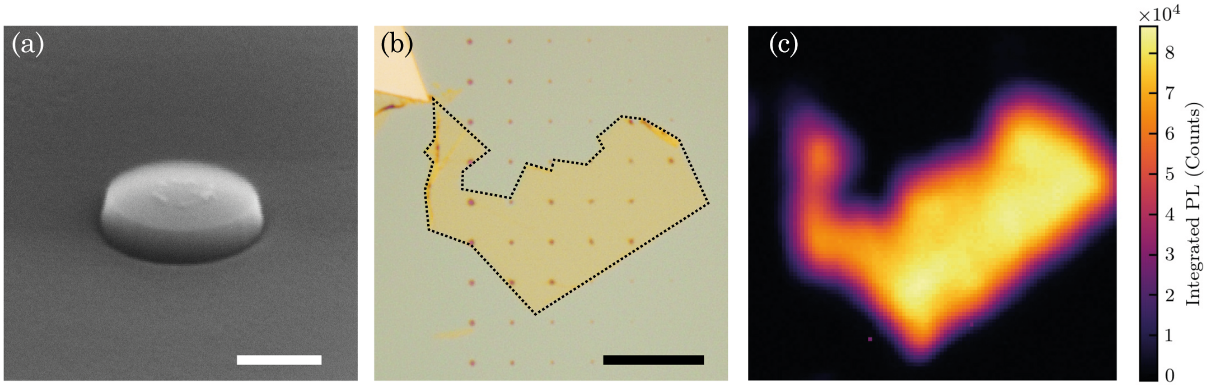


SUPPLEMENTARY FIGURE 6. (a) Electron microscopy tilted image of a $SiO_2$ nano-pillar ($h \approx$ 100 nm). Scale bar: 200 nm. (b) Bright field optical microscopy image of a monolayer $WSe_2$ (outlined by the dashed line) transferred on top of the array of $SiO_2$ nano-pillars. Scale bar: 10 $\mu$m. (c) Room temperature PL intensity map of the transferred monolayer $WSe_2$.

Supplementary Fig.7a shows a representative cryogenic PL spectrum of a $WSe_2$ SPE positioned on top of a $SiO_2$ nano-pillar (highlighted in grey). The localized emitter exhibits linearly polarized emission (Inset Supplementary Fig.7a), as expected from 2D in-plane dipole emitters. The SPE further shows saturation of the PL intensity under increasing excitation power (Supplementary Fig.7b), PL decay with $\tau = 5.8$ ns (Supplementary Fig.7c) and lower spectral stability compared to SPEs on GaP nano-antennas (Supplementary Fig.7d).

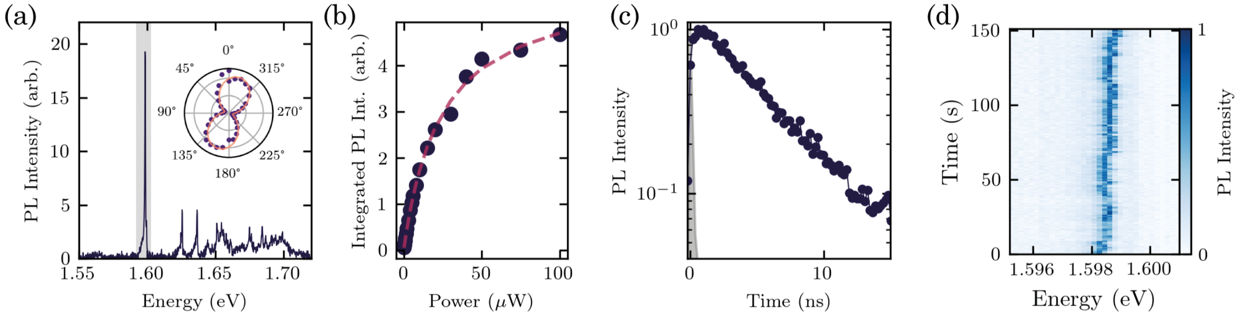


SUPPLEMENTARY FIGURE 7. (a) PL spectrum of a monolayer $WSe_2$ SPE on top of a $SiO_2$ nano-pillar, collected at a temperature of $T = 4$ K. Inset: polar plot of the linearly polarized emission for the SPE peak highlighted in grey. (b) Power saturation of the PL intensity under pulsed excitation at 638 nm and 80 MHz repetition rate. (c) Time resolved luminescence of the SPE localized on a $SiO_2$ nano-pillar (excitation pulse in grey). (d) Temporal stability of the PL emission from a SPE positioned on a $SiO_2$ nano-pillar ($h = 100$ nm).

## SUPPLEMENTARY NOTE V: STRAIN DEPENDENCE OF THE SINGLE PHOTON EMISSION

The nano-antenna geometry can be used to tailor the strain introduced in an atomically thin semiconductors as described in Ref.[6]. Supplementary Fig.8a shows the section along the x-axis, defined as in Fig.1 of the main text, of the height profile (black dashed line) of a $WSe_2$ monolayer on top of a dimer nano-antenna (in red) and the relative change in the $WSe_2$ conduction band potential ($V_{\mathrm{cb}}$). The tensile strain is maximized at the edges of the nano-pillars (in red) and correspond to a lowering of $V_{\mathrm{cb}}$, and directly a reduction of the band gap energy. This strain profile forms a deformation potential well which can trap excitons [6]. Where the 2D layer touches the substrate, strain becomes compressive and the $V_{\mathrm{cb}}$ is increased.

In Supplementary Fig.8b we show the position of different SPEs emission wavelengths (orange dots) and their average (purple), as a function of the nano-antenna radius. The increasing red-shift of the SPEs emission energy when on smaller radii nano-antennas is related to an increased tensile strain introduced in the 2D-$WSe_2$ membrane. These results confirm the impact of strain on the emission properties of strain-induced SPEs in two-dimensional $WSe_2$.

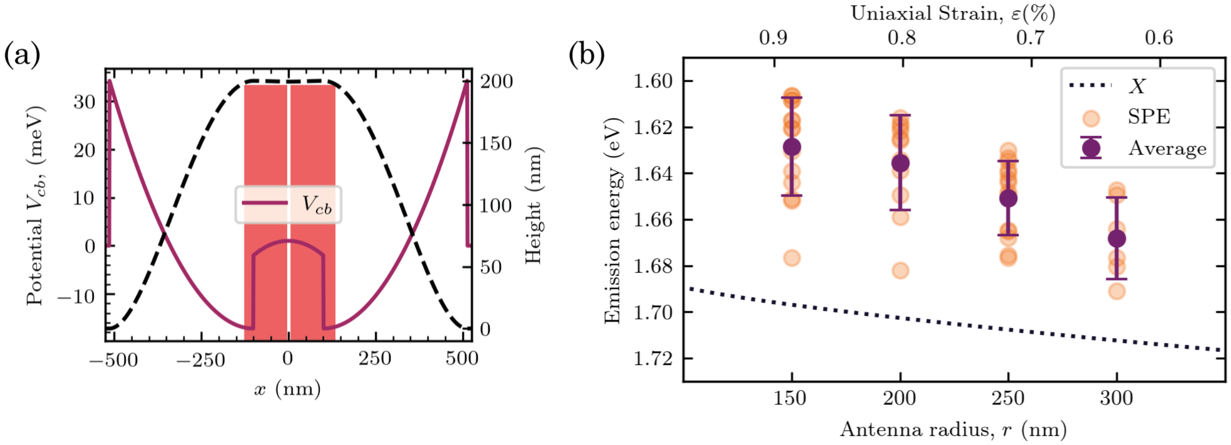


SUPPLEMENTARY FIGURE 8. (a) Cross-section along the x-axis (as in Figure 1 of the main text) of the strain-induced conduction band potential ($V_{\mathrm{cb}}$) modulation (in purple) calculated for the K valley of a monolayer $WSe_2$ as a function of x. The black dashed line shows the heigth profile for the same $WSe_2$ monolayer on top of the dimer nanoantenna (in red) [6]. (b) Emission energy of $WSe_2$ SPEs deposited on GaP nano-antennas with different radii (yellow dots), compared with the energy red-shift of the free exciton in $WSe_2$ ($X$, dashed line) as a function of the nano-antenna radius. In purple the average value, the error bars show the standard deviation. The dashed line is calculated from the unstrained value and obtained by interpolating the theoretical curve described in Ref.[6] with the experimental gauge of -49 meV/% under tensile strain for the $WSe_2$ exciton at room temperature from Ref.[7].

## SUPPLEMENTARY NOTE VI: COLLECTION AND QUANTUM EFFICIENCY OF THE SINGLE PHOTON EMISSION

The underlying quantum efficiency ($QE$) of a SPE under pulsed excitation can be estimated from the laser repetition rate and the number of detected photons [8]. If for each laser pulse we detect a photon, the $QE$ is 100% and the rate of photons detected matches that of the excitation laser repetition rate. We calibrated the collection efficiency by measuring the losses with a 725 nm laser. The values obtained from the calibration are listed in the table below. The values for the transmission of the linear polarizer, the spectrometer and the CCD efficiency are taken from the relative datasheet. We obtain a collection efficiency of the experimental setup of 0.56%.

| *Component* | *Transmission* |
|---|---|
| Optical components (Cryostat) | 50% |
| Linear polarizer | 78% |
| Single Mode fibre coupling | 2% |
| Spectrometer in-coupling | 90% |
| Spectrometer mirrors (x3) | $(97\%)^3$=91% |
| Grating Efficiency | 90% |
| CCD Quantum Efficiency | 98% |
| **TOTAL COLLECTION EFFICIENCY** | **0.56%** |

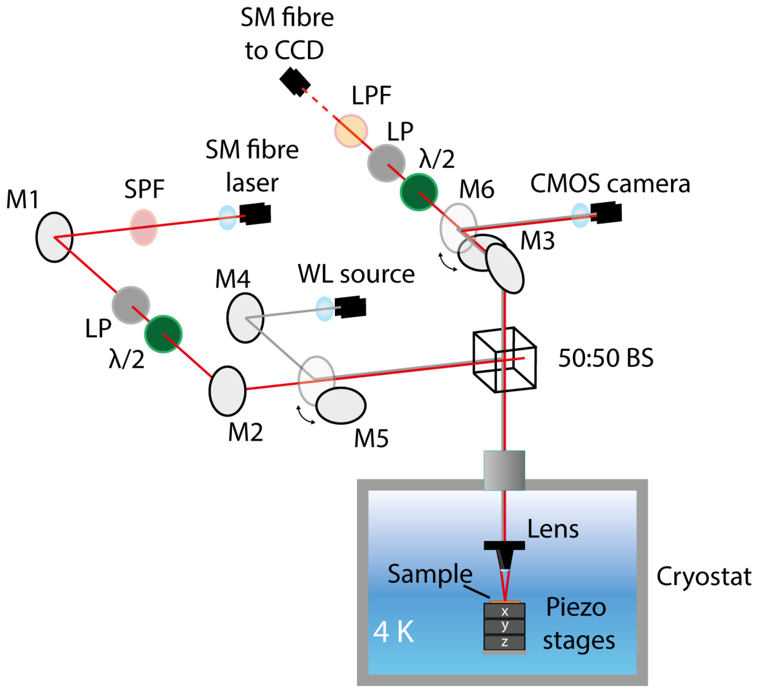


SUPPLEMENTARY FIGURE 9. Schematics of the experimental setup used to excite and collect the light from the SPEs placed in a bath cryostat at T = 4 K. SM: single mode fibre, SPF: short pass filter, M: mirror, LP: linear polarizer, $\lambda/2$: half-wave plate, WL source: white light source, BS: beam splitter, LPF: long pass filter.

From the FDTD simulations described in Supplementary Note I, we have estimated the cavity collection efficiency as the fraction of the total power radiated inside an objective with NA=0.64, the same used in our experiments. The estimated internal quantum efficiency ($QE$) is calculated as:

$$QE = \frac{I \cdot \eta_{\mathrm{exp}}^{-1} \cdot \eta_{\mathrm{coll}}^{-1}}{R} \quad (2)$$

where $I$ is the collected intensity from the SPE, $\eta_{\mathrm{exp}}$ is the collection efficiency of the experimental setup, $\eta_{\mathrm{coll}}$ is the collection efficiency from numerical simulations (given in Supplementary Note I), and $R$ is the laser repetition rate. In Supplementary Fig.10 we show the estimated internal quantum efficiency for $WSe_2$ SPEs positioned on both GaP dimer nano-antennas and $SiO_2$ nano-pillars, corrected for the total collection efficiency and for the corresponding laser repetition rate. For $SiO_2$, we obtain an average quantum efficiency of 4% while for SPEs on GaP an average of 21% with some SPEs reaching values as high as 86%, corresponding to an overall single photon emission rate of 69 MHz and, normalized to the collection efficiency, of a single photon rate of 5.5 MHz at the first lens.

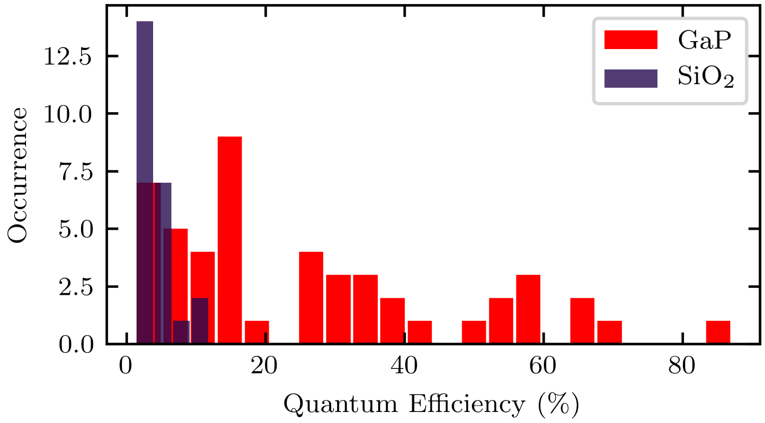


SUPPLEMENTARY FIGURE 10. Estimated internal quantum efficiency of the single photon emission in $WSe_2$ SPEs on GaP nano-antennas (red) and on $SiO_2$ nano-pillars (blue).

## SUPPLEMENTARY NOTE VII: PHOTOLUMINESCENCE DYNAMICS OF STRAIN-INDUCED $WSE_2$ SINGLE PHOTON EMITTERS

To obtain an analytical solution of the SPEs PL dynamics with a three level system, as discussed and depicted in Fig.3d in the main text, we use a reduced version of the model which can be solved analytically, given by the following equations:

$$\frac{dn_1}{dt} = -\frac{n_1}{\tau_1} + \frac{n_X}{\tau_2} \qquad \frac{dn_X}{dt} = -\frac{n_X}{\tau_2} \tag{3}$$

where $n_1$ and $n_X$ are the populations of the SPE and dark excitons, respectively. Here, $\tau_1 = (\Gamma_r + \Gamma_{nr})^{-1}$ is the recombination process of the SPE state giving rise to the luminescence, considering both radiative and non-radiative processes, while $\tau_2 = (\Gamma_{trap} + \Gamma_{nr}^{X})^{-1}$, composed of both trapping rate of a single exciton into the strain-induced potential and the non-radiative decay of the exciton population . We exclude from this model the quadratic Auger term and the saturation of the dot discussed in the main text. The analytical solution to the above equation system is given by:

$$n_1(t) = e^{-\frac{t}{\tau_1}}[n_1(0) + A(e^{\frac{t}{\tau_1}-\frac{t}{\tau_2}} - 1)] \tag{4}$$

where $A = n_X(0)\frac{\tau_1}{\tau_2 - \tau_1}$, and $n_X(0)$ and $n_1(0)$ are the initial conditions for each relative rate equation. The above equation is used to fit the experimental data and obtain the values of the rise time ($\tau_{rise} = \tau_2$) and decay time ($\tau_{decay} = \tau_1$) shown in the main text.
Supplementary Fig.11 shows additional PL decay from different SPEs, the PL spectra of which is shown in the figure inset, and fitted with the analytical solution of the model describe above. Under increasing power, the rise time reduces below the timing resolution of our experimental setup, and the PL decay becomes a single exponential profile.

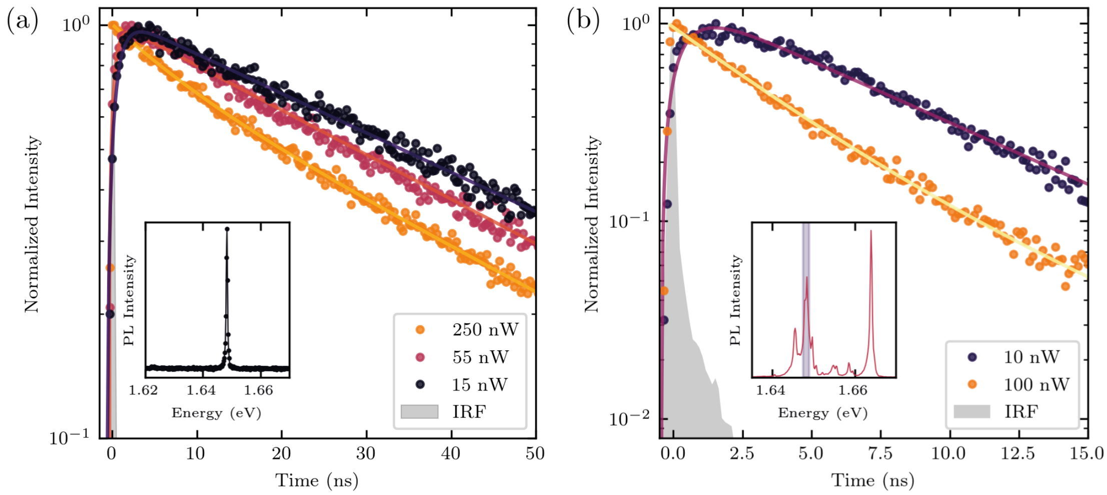


SUPPLEMENTARY FIGURE 11. (a) Power dependence of the PL lifetimes for the SPE shown in Figure 1 of the main text (see Inset). (b) Power dependence of the PL lifetimes for the side peaks of the SPE shown in Figure 3 of the main text (highlighted in grey in the Inset). In grey, the instrument response function (IRF).

[1] A. F. Koenderink, Single-Photon Nanoantennas, ACS Photonics **4**, 710 (2017).

[2] L. Sortino, P. G. Zotev, S. Mignuzzi, J. Cambiasso, D. Schmidt, A. Genco, M. Aßmann, M. Bayer, S. A. Maier, R. Sapienza, and A. I. Tartakovskii, Enhanced light-matter interaction in an atomically thin semiconductor coupled with dielectric nano-antennas, Nat. Commun. **10**, 5119 (2019).

[3] L. Novotny and B. Hecht, Principles of Nano-Optics (Cambridge University Press, Cambridge, 2006).

[4] C. Palacios-Berraquero, D. M. Kara, A. R.-P. Montblanch, M. Barbone, P. Latawiec, D. Yoon, A. K. Ott, M. Loncar, A. C. Ferrari, and M. Atatüre, Large-scale quantum-emitter arrays in atomically thin semiconductors, Nat. Commun. **8**, 15093 (2017).

[5] S. Kumar, M. Brotóns-Gisbert, R. Al-Khuzheyri, A. Branny, G. Ballesteros-Garcia, J. F. Sánchez-Royo, and B. D. Gerardot, Resonant laser spectroscopy of localized excitons in monolayer $WSe_2$, Optica **3**, 882 (2016).

[6] L. Sortino, M. Brooks, P. G. Zotev, A. Genco, J. Cambiasso, S. Mignuzzi, S. A. Maier, G. Burkard, R. Sapienza, and A. I. Tartakovskii, Dielectric Nanoantennas for Strain Engineering in Atomically Thin Two-Dimensional Semiconductors, ACS Photonics **7**, 2413 (2020).

[7] I. Niehues, R. Schmidt, M. Drüppel, P. Marauhn, D. Christiansen, M. Selig, G. Berghäuser, D. Wigger, R. Schneider, L. Braasch, R. Koch, A. Castellanos-Gomez, T. Kuhn, A. Knorr, E. Malic, M. Rohlfing, S. Michaelis de Vasconcellos, and R. Bratschitsch, Strain Control of Exciton-Phonon Coupling in Atomically Thin Semiconductors, Nano Lett. **18**, 1751 (2018).

[8] Y. Luo, G. D. Shepard, J. V. Ardelean, D. A. Rhodes, B. Kim, K. Barmak, J. C. Hone, and S. Strauf, Deterministic coupling of site-controlled quantum emitters in monolayer $WSe_2$ to plasmonic nanocavities, Nat. Nanotechnol. **13**, 1137 (2018).
